# Repeated cell sorting ensures the homogeneity of ocular cell populations expressing a transgenic protein

**DOI:** 10.1371/journal.pone.0265183

**Published:** 2022-03-25

**Authors:** Tsan-Chi Chen, Shu-Wen Chang

**Affiliations:** 1 Department of Ophthalmology, Far Eastern Memorial Hospital, New Taipei City, Taiwan; 2 Department of Ophthalmology, National Taiwan University Hospital, Taipei, Taiwan; Lobachevsky University, RUSSIAN FEDERATION

## Abstract

Transgenic proteins can be routinely expressed in various mammalian cell types via different transgenic systems, but the efficiency of transgene expression is constrained by the complex interplay among factors such as the temporal consistency of expression and compatibility with specific cell types, including ocular cells. Here, we report a more efficient way to express an enhanced green fluorescent protein (EGFP) in human corneal fibroblasts, corneal epithelial cells, and conjunctival epithelial cells through a lentiviral expression system. The relative transducing unit criterion for EGFP-expressing pseudovirions was first determined in HEK-293T cells. Homogeneous populations of EGFP-positive and EGFP-negative cells could be isolated by cell sorting. The half-maximal inhibitory concentration (IC_50_) value for puromycin was calculated according to viability curves for each cell type. The results revealed that cell types differed with respect to EGFP expression efficiency after transduction with the same amount of EGFP-encoding pseudovirions. Using a cell sorter, the homogeneity of EGFP-positive cells reached >95%. In the initial sorting stage, however, the efficiency of EGFP expression in the sorted cells was noticeably reduced after two rounds of sequential culture, but repeated sorting for up to four rounds yielded homogeneous EGFP-positive human corneal fibroblasts that could be maintained in continuous culture *in vitro*. The sorted EGFP-positive cells retained their proper morphology and cell type-specific protein expression patterns. Puromycin resistance was found to depend on cell type, indicating that the IC_50_ for puromycin must be determined for each cell type to ensure the isolation of homogeneous EGFP-positive cells. Taken together, repeated cell sorting is an efficient means of obtaining homogeneous populations of ocular cells expressing a transgenic protein during continuous culture without the potential confounding effects of antibiotics.

## Introduction

Transgenic expression of a protein in various cell types is commonly used to change the intracellular expression of the protein toward the goal of determining its function(s) [1–4]. Such analyses can provide information concerning the route of intracellular transport, the ability to facilitate cellular transformation to yield a malignant state [5], and the production of functional recombinant proteins [6]. These types of studies require an efficient system for transgene expression.

Various types of genetic elements, such as small interfering RNAs and plasmids that may encode diverse molecules, can be transferred into mammalian cells by various techniques including electroporation (*in vitro* or *in vivo*) [7], lipid-based reagents [8], and virus-based vectors [3, 9, 10]. Regardless of the delivery process, mammalian cells often suffer post-electroporation damage [11], chemical insults [12], or off-target integration of the transgene (in the case of lentiviral transduction) [13]. Based on the requirements of any mammalian-cell experimental system, each transfection method may have numerous factors that affect expression efficiency as well as the persistence of the transgene(s).

To track transgene expression in mammalian cells that have been transfected with a vector, various fluorescent proteins, including enhanced green fluorescent protein (EGFP), yellow fluorescent protein, and red fluorescent protein, are frequently encoded by the transgene open-reading frame by gene fusion [14, 15] or an internal ribosome entry site–driven bicistronic expression system [3, 16, 17]. The expression and intracellular dynamics of the fusion protein can be monitored by fluorescence microscopy and/or flow cytometry. Therefore, transgene-expressed fluorescent proteins are widely used as reporters.

No matter which method is used for transgene delivery in target cells, the efficiency of transgene expression is the primary concern. Therefore, researchers have carried out numerous studies to improve expression efficiency by modifying one or more of the following three experimental aspects: the reaction conditions during electroporation [18], the composition of lipid-based transfection systems [19], and the type of vector in viral transduction systems [20].

The second major concern is retention of a high transgene copy number over numerous cell-culture passages while limiting cytotoxicity. To isolate mammalian cells that stably express a transgene, several antibiotic-resistance genes have been designed and integrated into expression vectors for mammalian cells, *e*.*g*., genes for resistance to bleomycin, geneticin, hygromycin, puromycin, and zeocin [21]. However, any antibiotic can potentially trigger cytotoxicity over time and thus affect transgene expression [22], a situation from which transgenic cells may not recover.

Fortunately, cell sorting provides an efficient way to isolate a homogeneous cell population without antibiotic selection [23]. Indeed, the cell-sorting process has been streamlined to enable the efficient isolation of homogeneous cell populations based on diverse cellular characteristics such as size, morphology, and intensity of fusion protein–emitted fluorescence. Therefore, ever-increasing numbers of researchers have chosen this more experimentally effective means of selecting and isolating various types of transgenic cells, including immune cells [24], stem cells [25], and tumor cells [26].

Whether transgene expression is mediated by pseudoviral transduction [27] or liposome delivery [28], it is crucial to retain the intrinsic characteristics in the transduced cells. The cell characteristics can be verified by monitoring the abundance of constitutively expressed proteins, *e*.*g*., Thy-1 [29] and alpha-smooth muscle actin (α-SMA) in human corneal fibroblasts (HCFs) and myofibroblasts [30, 31], cytokeratin-13 (CK13) in human conjunctival epithelial cells (HCjEs) [32], and zonula occludens-1 (ZO-1) in both HCjEs and human corneal epithelial cells (HCnEs). In addition, it is also critical to monitor the expression of constitutively expressed proteins involved in cell viability such as B-cell lymphoma-extra-large (Bcl-xL) [33], focal adhesion kinase (FAK), and phosphatase and tensin homolog (PTEN) [34].

Herein, we demonstrate how cell sorting increases the fluorescence intensity emitted by ocular cells that have been transduced via a lentiviral transgene-delivery system. We discuss the potential benefit of a cell sorter–based means of maintaining the efficiency of transgene expression in ocular cells without the potential negative effects of a selecting antibiotic(s).

## Materials and methods

### Human cells

The human ocular cells included primary HCFs, HCnEs, and HCjEs. The human kidney cell line HEK-293T was used to prepare the lentiviral EGFP-encoding pseudovirions. The various cell types were cultured under the conditions described below.

#### Primary HCFs

The need for consent for the use of human donor corneal rims was waived by the Institutional Research Ethics Review Committee, Far Eastern Memorial Hospital, New Taipei City, Taiwan (Protocol No. FEMH-101002-F, and FEMH-108077-W). Briefly, primary HCFs were directly isolated from residual corneas during penetrating keratoplasty surgery according to established isolation protocols [14, 31, 35], whose personal identifiers and patient information were delinked from the specimens. The isolated HCFs that had been passaged two or three times were stored in liquid nitrogen. The stored HCFs were randomly used and cultured at 37°C in DMEM (Life Technologies, Grand Island, NY) containing 10% heat-inactivated fetal bovine serum (FBS; Life Technologies), 100 U/ml penicillin G (Sigma-Aldrich, St. Louis, MO), and 100 μg/ml streptomycin sulfate (Sigma-Aldrich) in humidified air with 5% CO_2_. HCFs were passaged by trypsinization with 0.05% trypsin-EDTA (Life Technologies), and the culture medium was refreshed every 2 to 3 days.

#### HCnEs

HCnEs, an immortalized corneal cell line from the parental line HCE-2 (CRL-11135; ATCC, Manassas, VA), were passaged in DMEM/F-12 containing 10% FBS, 1% non-essential amino acids (Life Technologies), 100 U/ml penicillin, and 100 μg/ml streptomycin at 37°C under 5% CO_2_. The culture medium was refreshed every 2 to 3 days.

#### HCjEs

HCjEs, an immortalized human conjunctival cell line (CCL-20.2; ATCC), were passaged in M199 medium (Life Technologies) containing 10% FBS, 100 U/ml penicillin, and 100 μg/ml streptomycin at 37°C under 5% CO_2_. The culture medium was refreshed every 2 to 3 days.

#### HEK-293T cells

Human embryonic kidney cells from a cell line HEK-293T (CRL-11268; ATCC) were passaged in DMEM containing 10% FBS, 100 U/ml penicillin, and 100 μg/ml streptomycin at 37°C under 5% CO_2_. The culture medium was refreshed every 2 to 3 days.

### EGFP expression system

#### EGFP plasmid

The expression plasmid pAS2.EGFP.puro, which encodes EGFP, was obtained from the National RNAi Core Facility at Academia Sinica, Taiwan. The puromycin-resistance domain was included in the EGFP plasmid to differentiate between EGFP-positive and EGFP-negative cells.

#### Production of EGFP-encoding pseudovirions

To express the lentiviral EGFP, pseudovirions encoding EGFP were produced according to a published procedure with appropriate modifications [3]. Briefly, 1.0 × 10^6^ HEK-293T cells were seeded in a 10-cm culture dish with antibiotic-free DMEM containing 10% FBS at 37°C under 5% CO_2_. On the following day, the cells were replenished with fresh antibiotic-free DMEM containing 10% FBS and incubated for 16 h with 300 μl of a transfection mixture in Opti-MEM medium containing 7.5 μg pAS2.EGFP, 6.75 μg pCMVΔR8.91, and 0.75 μg pMD.G with 60 μl X-treme GENE HP DNA Transfection Reagent (Roche Applied Science, Indianapolis, IN). Subsequently, the transfected cells were transferred into 10 ml antibiotic-free DMEM containing 10% FBS and 1% bovine serum albumin (Alpha Biochemistry, Taoyuan City, Taiwan) for 2 days. After removing residual dead cells through filtration with a 0.22-μm filter, the EGFP pseudovirion-containing supernatant was collected and stored at –80°C until used for titration and transduction of ocular cells.

#### Titration and transduction of cells with EGFP-encoding pseudovirions

Expression of lentiviral EGFP in ocular cells followed the standard protocol of the National RNAi Core Facility at Academia Sinica, Taiwan, to estimate the transduction efficiency of the EGFP-encoding pseudovirions by titration in HEK-293T cells. From a 1-ml stored supernatant containing the pseudovirions, a series of 10-fold dilutions was used to directly transduce 2.0 × 10^5^ HEK-293T cells in each well of a 6-well plate in antibiotic-free DMEM containing 10% FBS and 8 μg/ml polybrene (Sigma-Aldrich). The lentiviral EGFP titer was calculated at 3 days post-transduction by flow cytometry according to the standard formula: titer = [(F × Cn)/V] × DF, where F is the frequency of EGFP-positive cells determined by flow cytometry, Cn is the total number of transduced cells, V is the volume of the inoculum, and DF is the virus dilution factor. The final transduction titer was represented as the relative transducing unit (R.T.U.) per milliliter. For various experiments, the criterion of R.T.U. was used to determine the appropriate amount of packaged EGFP-encoding pseudovirions for transduction of primary HCFs, HCnEs, HCjEs, and HEK-293T cells.

### Flow cytometry

All cells were passaged and cultivated in 10-cm cell-culture dishes under their specific culture conditions described above. At 80–90% confluency, cells were harvested by trypsinization with 0.05% trypsin-EDTA. After washing once with phosphate-buffered saline (137 mM NaCl, 2.7 mM KCl, 10 mM Na_2_HPO_4_, 2 mM KH_2_PO_4_, pH 7.4), the cells were resuspended in 5 ml of their specific culture medium and filtered through a 50-μm nylon mesh. Then, the cells were directly analyzed with a MoFlo XDP High-Performance Cell Sorter (Beckman Coulter, Fullerton, CA).

### Cell sorting

According to the dot plots of FL1 (green channel) vs FL3 (red channel) as determined with the MoFlo XDP High-Performance Cell Sorter, both EGFP-positive and EGFP-negative cells were gated and separately collected into a 10-cm culture dish. After complete adhesion in the culture dish, the collected cells were incubated in their specific culture medium until the next passage. Routine passaging continued until more than 95% of cells were EGFP-positive in two consecutive passages.

### Cell proliferation

To compare the rate of proliferation of the lentiviral EGFP-positive and EGFP-negative cells, all the cells were evaluated by a cell proliferation assay with the Cell Counting Kit-8 (CCK-8) reagent (Dojindo Laboratories, Kumamoto, Japan). Briefly, 5.0 × 10^3^ cells were cultivated individually in each well of a 96-well plate. On the day after complete adhesion, the appropriate culture medium was refreshed for the cells in each well, and this corresponded to time zero of the assay. At 0 h, 24 h, and 48 h, 10 μl CCK-8 reagent was directly added into each well and subsequently incubated at 37°C for 2 h. Using a spectrophotometer, the absorbance of the samples was measured at 450 nm against a reference at 690 nm (OD_450_ –OD_690_) as the baseline for subsequent experiments.

### Cell morphology

To compare the morphology of each pure EGFP-positive cell type stored in liquid nitrogen, the stored cells were thawed, seeded, and allowed to attach completely in a 12-well culture plate. Cell morphology and EGFP images were captured separately in the bright field and fluorescence field using an inverted fluorescence microscope with the imaging software cellSens Entry 1.18 (Olympus, Tokyo, Japan).

### Immunoblotting

To determine the relative levels of constitutively expressed proteins in the sorted cells, the cell lysates were harvested after being cultured in a 6-cm culture dish as described above. Briefly, the cells were washed with phosphate-buffered saline, disrupted in lysis buffer (150 mM NaCl, 1% NP-40, 0.2% SDS, 0.5% sodium deoxycholate, 50 mM Tris-HCl, pH 7.4) containing a tablet of complete EDTA-free protease inhibitor cocktail (Cat. No. 04693132001; Roche Applied Science). The concentration of total protein in each cell lysate was calculated by the BCA Protein Assay kit (Pierce, Rockford, IL). According to the expression of each target protein in the sorted cells, 5 μg of each cell lysate was mixed with 3× sample buffer (180 mM Tris-HCl, pH 6.8; 30% glycerol; 6% SDS; 3.75% β-mercaptoethanol) and subjected to SDS-PAGE. The proteins in each gel were transferred to a polyvinylidene difluoride membrane (Millipore, Billerica, MA). After blocking nonspecific binding with 5% bovine serum albumin in TBST buffer (20 mM Tris-HCl, pH 7.5; 500 mM NaCl; 0.1% Tween-20) for 1 h, duplicate membranes were separately incubated with a primary antibody specific for α-SMA (ab32575; rabbit monoclonal; Abcam, Waltham, MA), β-actin (A1978; mouse monoclonal; Sigma-Aldrich), Bcl-xL (ab32370; rabbit monoclonal; Abcam), CK13 (sc-101460; mouse monoclonal; Santa Cruz Biotechnology, Santa Cruz, CA), GFP (sc-8334; rabbit polyclonal; Santa Cruz Biotechnology), PTEN (ab32199; rabbit monoclonal; Abcam), Thy-1 (ab133350; rabbit monoclonal; Abcam), or ZO-1 (ab96587; rabbit monoclonal; Abcam) at 4°C overnight. After the membranes were washed three times with TBST buffer for 20 min each, they were incubated with the appropriate horseradish peroxidase–conjugated secondary antibody against rabbit (ab6802; donkey polyclonal; Abcam) or mouse (ab6820; donkey polyclonal; Abcam) for 1 h. After washing three times with TBST buffer for 20 min each, each blot was developed with Immobilon Western Chemiluminescent HRP substrate (WBKLS0500; Millipore), and images were captured using a Fujifilm LAS-4000 imaging system (FUJIFILM, Tokyo, Japan). Finally, the intensity of immunopositive bands was determined and analyzed using Multi Gauge software (FUJIFILM).

### Treatment of cells with puromycin

To compare the sensitivity of the lentiviral EGFP-positive and EGFP-negative cells to antibiotics, all cells that had been treated with puromycin were also evaluated by the cell proliferation assay with CCK-8 reagent, as described above. Briefly, 5.0 × 10^3^ cells were cultivated individually in each well of a 96-well plate. On the day after complete adhesion, the appropriate culture medium was refreshed for the cells in each well (time zero); the medium contained 3.16-fold serial dilutions of puromycin, ranging from 0.01 to 316 μg/ml. At 48 h, 10 μl CCK-8 reagent was directly added into each well and subsequently incubated at 37°C for 2 h. Cell proliferation was calculated according to the following formula: cell viability (%) = [(OD_450_ –OD_690_) in puromycin-treated group–(OD_450_ –OD_690_) at baseline]/[(OD_450_ –OD_690_) in puromycin-untreated group–(OD_450_ –OD_690_) at baseline] × 100%. Baseline was determined in cell-free culture medium. The half-maximal inhibitory concentration (IC_50_) was calculated using a formula derived from each cell viability curve using Prism 8 software (GraphPad Inc., San Diego, CA).

### Statistical analysis

Each experiment was performed independently as described in the figure legends. The differences among groups were evaluated using the Student’s *t*-test or one-way analysis of variance followed by Tukey’s honestly significant difference post-hoc test as described in the figure legends. *P* values less than 0.05 were considered statistically significant.

## Results

### Differences in transgene expression efficiency among various ocular cells transduced with lentiviral EGFP

**Fig 1:**
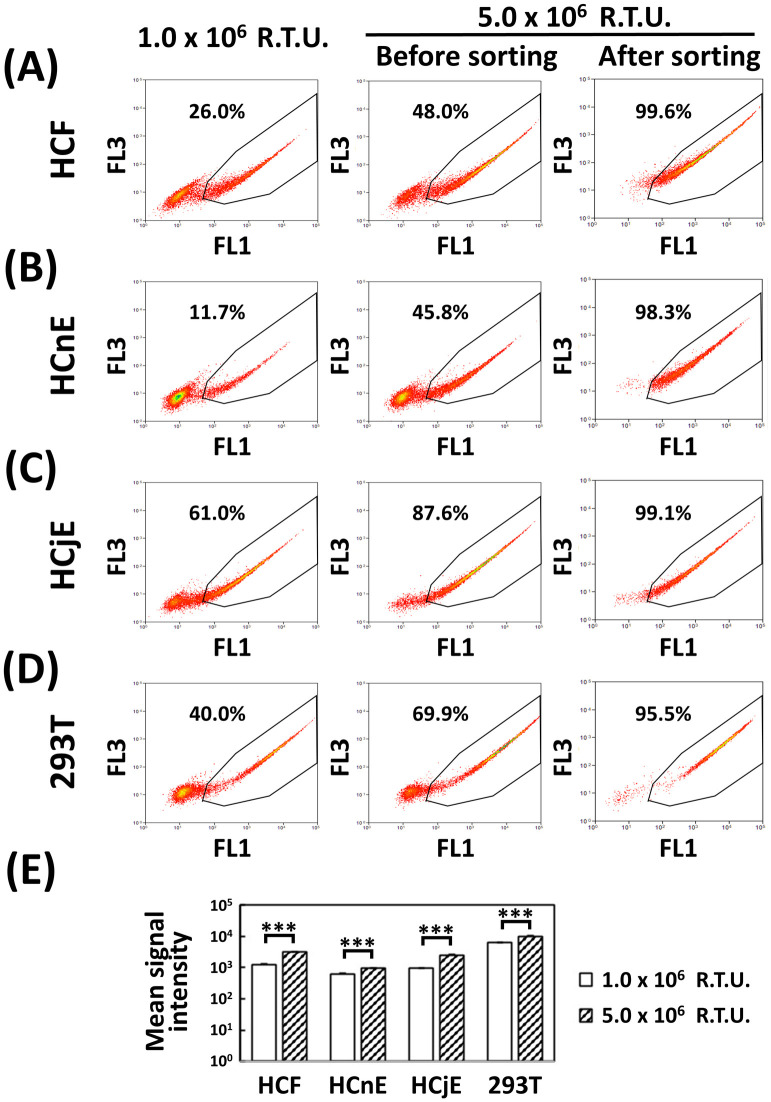
Differences in expression efficiency after transduction of various ocular cell types with EGFP-encoding pseudovirions. Each cell type was seeded into a 10-cm culture dish: (A) 2.0 × 10^5^ HCFs, (B) 2.0 × 10^6^ HCnEs, (C) 2.0 × 10^6^ HCjEs, and (D) 2.0 × 10^6^ HEK-293T cells. At 24 h post-seeding, each cell type was treated with EGFP-encoding pseudovirions at 1.0 × 10^6^ R.T.U. or 5.0 × 10^6^ R.T.U. for 16 h. Subsequently, the culture media were refreshed for all transduced cells until >90% confluency was attained. Cell sorting was used to analyze the transduced cells with 1.0 × 10^6^ R.T.U. (left panels) or 5.0 × 10^6^ R.T.U. (middle panels). The right-most panels present data acquired after sorting of the cells transduced with 5.0 × 10^6^ R.T.U. The percentages represent the homogeneity of EGFP-positive cells in the gating regions. (E) The mean signal intensity was the average signal intensity of the gated EGFP-positive cells in the FL1 channel (S1 Fig). The analysis of transduction of each cell type with two different titers of EGFP pseudovirions was repeated three times with similar trends. The shown data represent the results of one of the experiments. Data are expressed as the mean ± S.D. from three independent experiments. Differences between two different titers of EGFP pseudovirions were analyzed with the Student’s *t*-test (***P < 0.001). FL1 is the green channel, and FL3 is the red channel. R.T.U., relative transducing unit.

To examine EGFP expression efficiency in ocular cells, EGFP-encoding pseudovirions were titrated in HEK-293T cells. These pseudovirions were used to transduce HCFs (Fig 1A), HCnEs (Fig 1B), HCjEs (Fig 1C), and HEK-293T cells (Fig 1D). Using a total of 1.0 × 10^6^ R.T.U., 40.0% of the transduced HEK-293T cells expressed EGFP, a percentage similar to that obtained from the titration experiment. For the three ocular cell types transduced with the same pseudovirion load, EGFP expression increased by the following percentages relative to HEK-293T cells: 26.0% for HCFs, 11.7% for HCnEs, and 61.0% for HCjEs. The use of a 5-fold greater number of pseudovirions to transduce the cells resulted in only a modest increase in the percentage of EGFP-positive cells, *i*.*e*., 48.0% for HCFs, 45.8% for HCnEs, 87.6% for HCjEs, and 69.9% for HEK-293T cells. The corresponding changes in intracellular EGFP fluorescence (*i*.*e*., expression) were significant, however, namely 2.47 fold for HCFs, 1.50 fold for HCnEs, 2.52 fold for HCjEs, and 1.58 fold for HEK-293T cells (Fig 1E and S1 Fig).

### Efficiency of cell sorting to isolate a homogeneous population of EGFP-positive ocular cells

To enhance EGFP expression in the ocular cells, a cell sorter with single-cell mode was used to isolate the EGFP-positive cells after pseudovirion transduction with a high titer for each cell type and corresponding cell number (Fig 1, middle panels). The percentage of EGFP-positive cells before sorting was 48.0% for HCFs, 45.8% for HCnEs, 87.6% for HCjEs, and 69.9% for HEK-293T cells. However, a single round of flow cytometry with single-cell mode increased the homogeneity of EGFP-positive cells to >95% (Fig 1, right panels).

### Repeated cell sorting increases the homogeneity of EGFP-positive ocular cells

**Fig 2:**
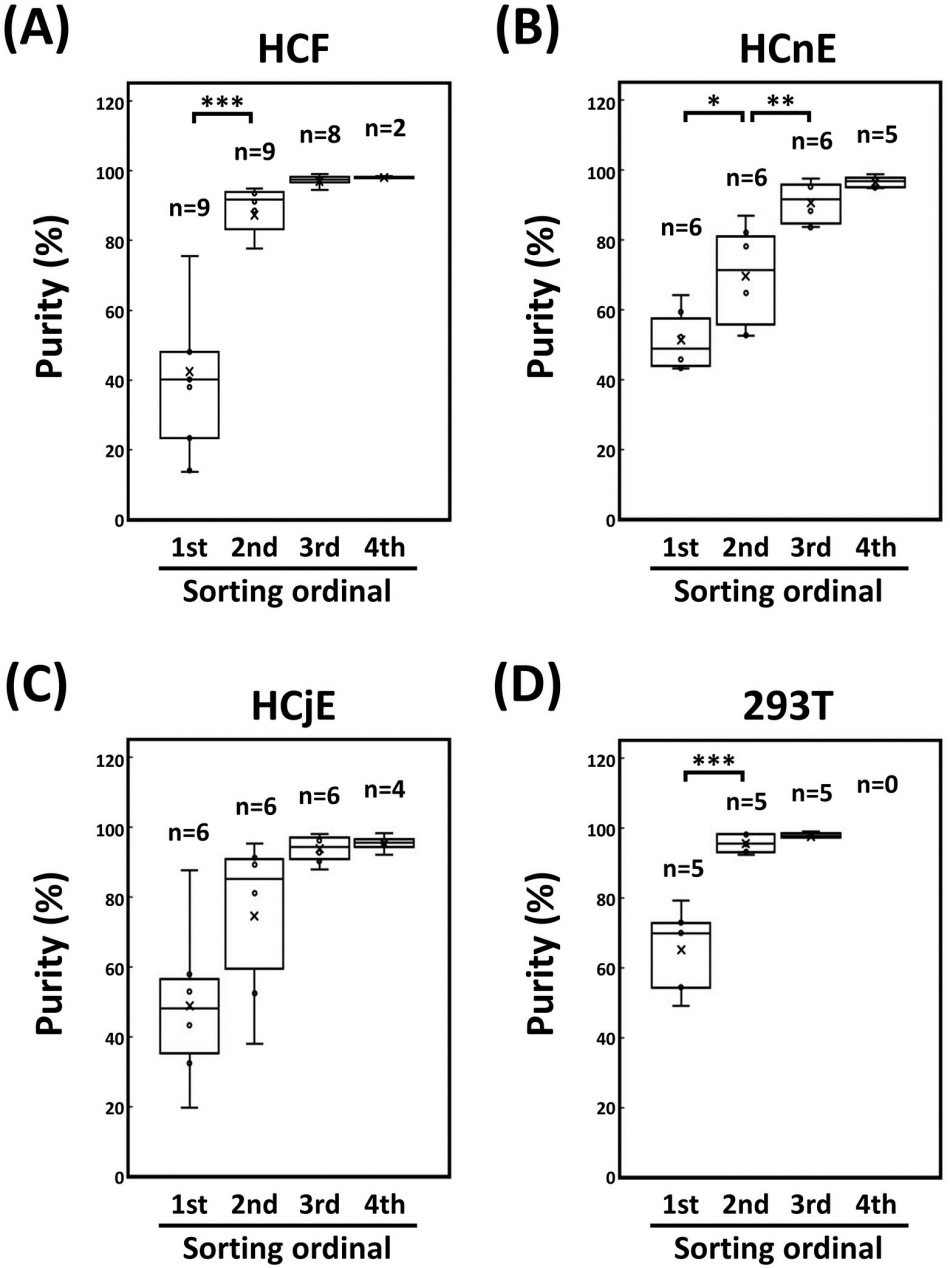
Purity of the EGFP-positive cells after sequential passaging and sorting. Each cell type was seeded into a 10-cm culture dish: (A) 2.0 × 10^5^ HCFs, (B) 2.0 × 10^6^ HCnEs, (C) 2.0 × 10^6^ HCjEs, and (D) 2.0 × 10^6^ HEK-293T cells. At 24 h post-seeding, each cell type was incubated EGFP-encoding pseudovirions at 5.0 × 10^6^ R.T.U. for 16 h. Subsequently, the culture media were refreshed for each of the transduced cells until >90% confluency was attained. The transduced cells were analyzed and sorted with a cell sorter at the indicated rounds. All rounds of cell sorting were repeated until the proportion of EGFP-positive cells was >95% in two successive passages. Box-whisker plots were drawn to show the proportion of sorted cells expressing EGFP. The n values on the box-whisker plots represent the number of different donor corneas from which HCFs were harvested (A). The n values on the box-whisker plots represent the number of experiments performed at the indicated sorting times for HCnEs (B), HCjEs (C), and HEK-293T cells (D). Horizontal lines represent the median, boxes denote the two inner quartiles, and whisker bars show the maximum and minimum values for expression efficiency. S2 Fig lists all the proportions of the EGFP-positive cells during cell sorting at the indicated rounds. Differences in the purity of EGFP-positive cells were analyzed separately by one-way analysis of variance followed by Tukey’s honestly significant difference post-hoc test (*P < 0.05; **P < 0.01; ***P < 0.001).

For the aforementioned sorted EGFP-positive cells (*i*.*e*., >95% homogeneity; Fig 1), the percentage of EGFP-positive cells decreased after two additional rounds of sequential culture to amplify the number of cells. For example, transduction of primary HCFs with EGFP-encoding pseudovirions yielded only 42.5± 25.6% EGFP-positive cells (Fig 2A) compared with >95% purity after the first sorting. However, the percentage of EGFP-positive HCFs decreased to 87.3 ± 11.5% after passaging twice. Therefore, the sorting process was repeated to isolate a homogeneous population of persistent EGFP-positive HCFs until the proportion of those cells was >95% in two successive passages.

### Persistent EGFP expression in ocular cells requires up to four rounds of cell sorting

In addition to HCFs, both HCnEs (Fig 2B) and HCjEs (Fig 2C) were subjected to repeated cell sorting to assess the validity of this approach for purifying EGFP-positive cells. HEK-293T cells were included as a non-ocular-cell control in addition to their use for packaging EGFP-encoding pseudovirions (Fig 2D). All these cells were also transduced with the EGFP-encoding pseudovirions to yield 51.3 ± 8.9% EGFP-positive HCnEs, 49.0 ± 23.5% EGFP-positive HCjEs, and 65.1 ± 12.8% EGFP-positive HEK-293T cells. After sequentially passaging twice, however, the percentage of EGFP-positive cells decreased from >95% purity to 69.6 ± 15.0% for HCnEs, 74.6 ± 23.7% for HCjEs, and 95.5 ± 2.8% for HEK-293T cells. Based on the default criterion mandating >95% EGFP-positive cells in two consecutive passages, we found that up to four rounds of cell sorting were required to yield a homogeneous population of EGFP-positive ocular cells.

### Potential effect of transgenic EGFP on cell proliferation

**Fig 3:**
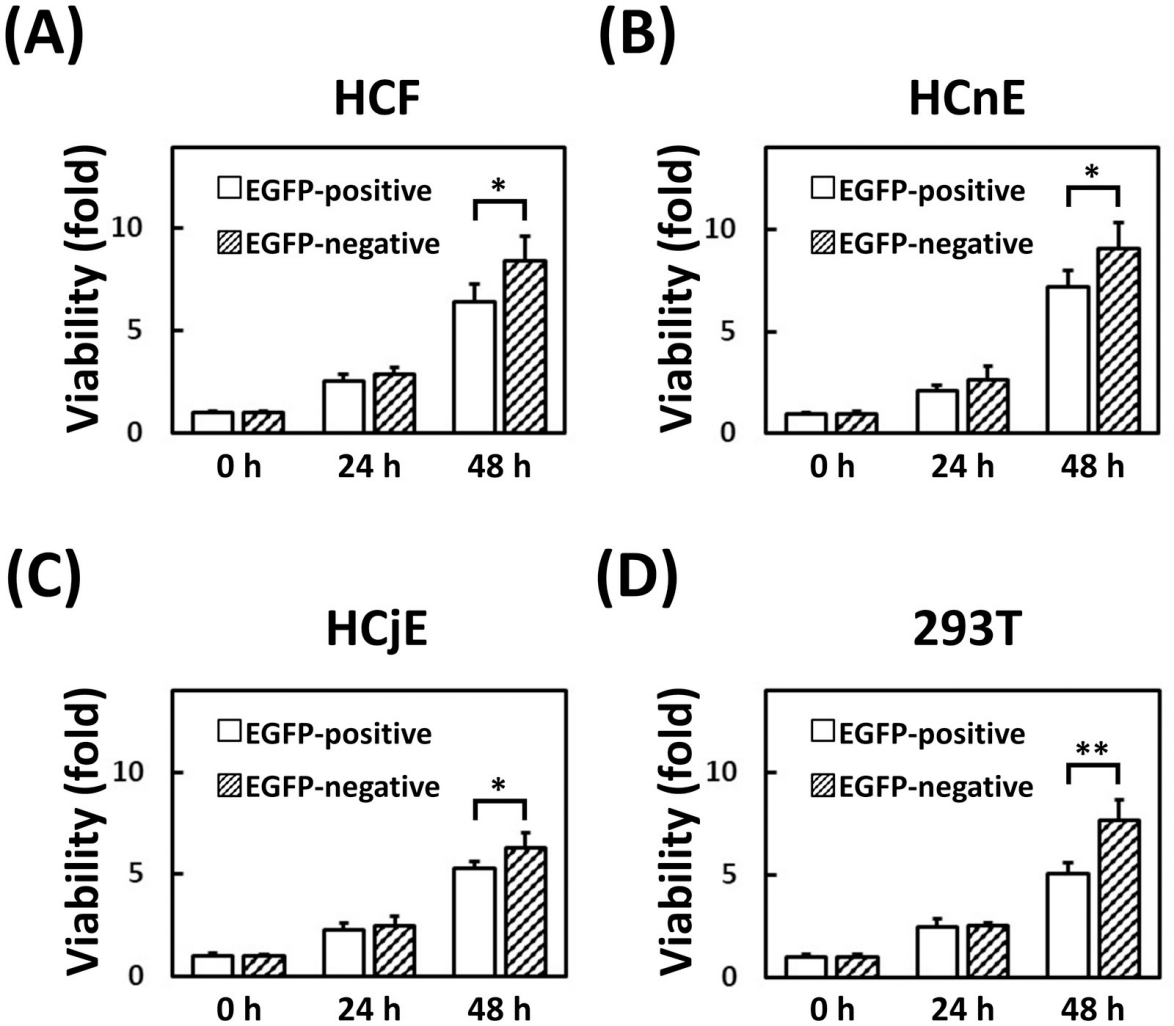
Viability of the sorted EGFP-expressing cells. Each cell type was seeded into individual wells of a 96-well plate: (A) 2.0 × 10^3^ HCFs, (B) 2.0 × 10^4^ HCnEs, (C) 2.0 × 10^4^ HCjEs, and (D) 2.0 × 10^4^ HEK-293T cells. At 24 h post-seeding as the starting time (0 h), cell viability was analyzed at 0, 24, and 48 h by CCK-8 reagent. Data are expressed as the mean ± S.D. from three independent experiments. The upper bars represent the S.D. of the mean. Differences in the viability of EGFP-positive and EGFP-negative cells were analyzed with the Student’s *t*-test (*P < 0.05; **P < 0.01).

To assess any potential effect of transgenic EGFP on cell proliferation, the viability of the three ocular cell types and non-ocular cells was analyzed with a proliferation assay for EGFP-positive and EGFP-negative cells (Fig 3). Both EGFP-positive and EGFP-negative cells were synchronized by seeding an equal number of sorted cells in the same sized culture dish until the proportion of those cells was >95% in two successive passages. At 48 h, the proliferation rate of EGFP-positive cells was significantly lower than that of the EGFP-negative cells, *i*.*e*., 6.43 ± 0.89 vs 8.45 ± 1.18 fold for primary HCFs (*P* = 0.038), 7.15 ± 0.81 vs 9.06 ± 1.25 fold for HCnEs (*P* = 0.009), 5.32 ± 0.34 vs 6.32 ± 0.69 fold for HCjEs (*P* = 0.044), and 5.04 ± 0.55 vs 7.65 ± 1.02 fold for HEK-293T cells (*P* = 0.009).

### Cell sorting might induce fibroblast-like characteristics in EGFP-positive cells

**Fig 4:**
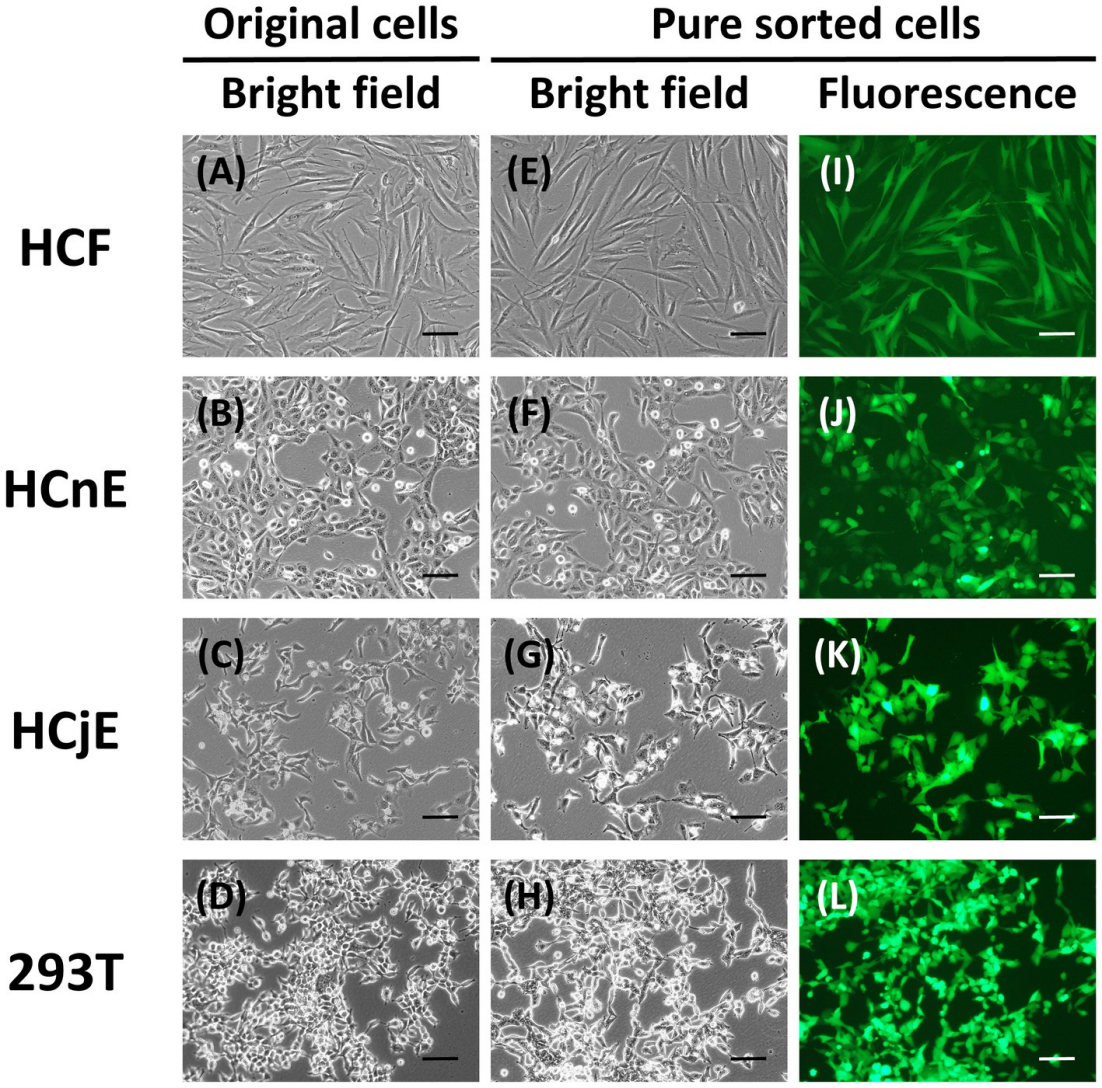
Morphology of the sorted EGFP-expressing cells. (A-D) The morphology of the primary HCFs, HCnEs, HCjEs, and 293T cells without transduction by EGFP-expressing pseudovirions but after repeated cell sorting was captured under a bright field microscope at 100× magnification. (E-H) The morphology of the sorted EGFP-positive cells was captured using a light microscope at 100× magnification. (I-L) In the same fields, fluorescence images of the sorted EGFP-positive cells were captured using a fluorescence microscope. This experiment was performed three times with similar results. Scale bars, 100 μm. Other images for cell morphology are available at Figshare (DOI: 10.6084/m9.figshare.18134096).

**Fig 5:**
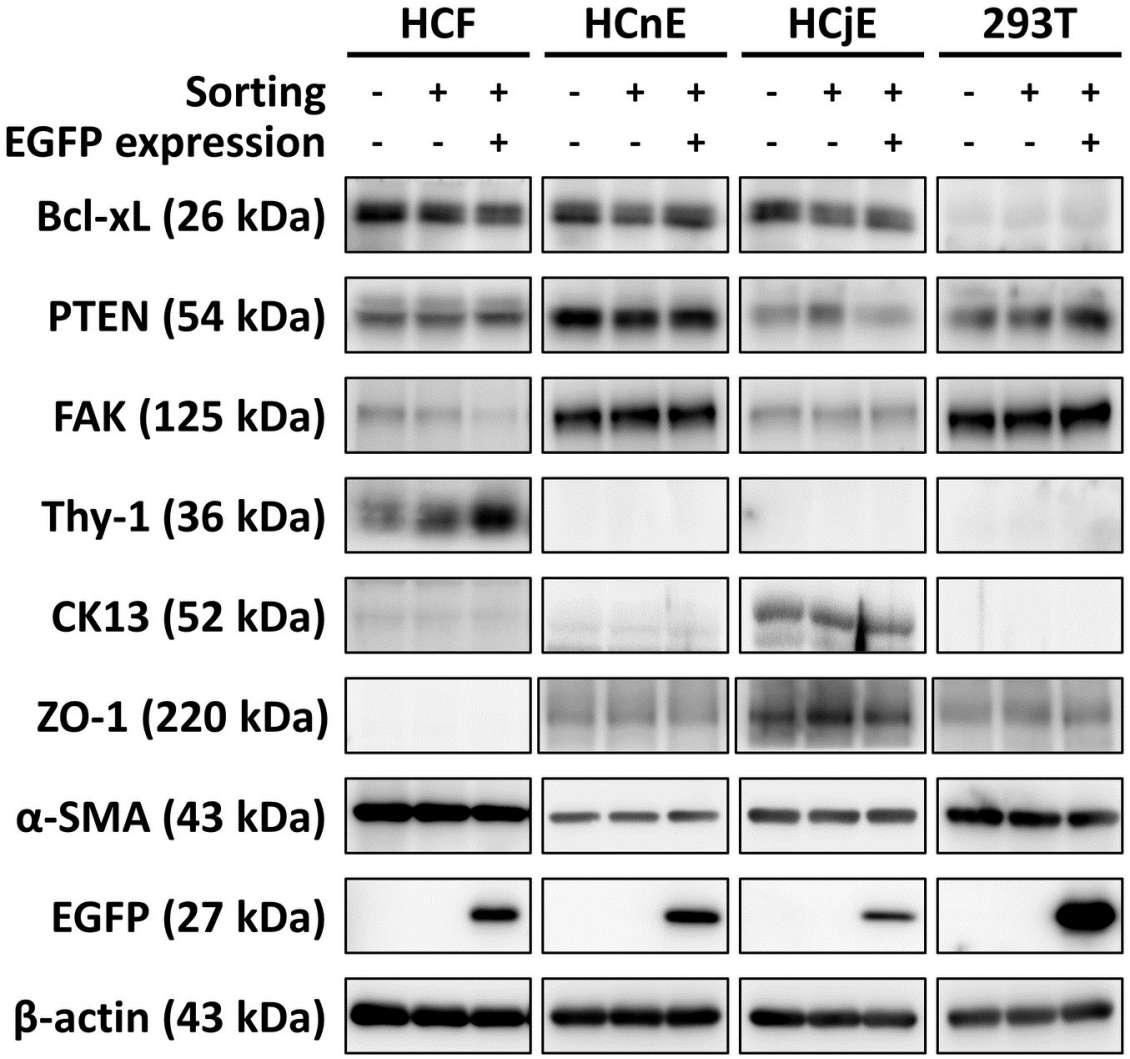
Alteration of cell type-specific expression proteins in the sorted EGFP-expressing cells. Non-transduced cells, sorted EGFP-negative cells, and sorted EGFP-positive cells of the four cell types were separately cultured in 6-cm dishes, and the cell lysates were prepared for immunoblotting analysis of cell type-specific protein profiles. This experiment was performed three times with similar results. Bcl-xL, B-cell lymphoma-extra large; PTEN, phosphatase and tensin homolog; FAK, focal adhesion kinase; CK13, cytokeratin-13; ZO-1, zonula occludens-1; α-SMA, alpha-smooth muscle actin; EGFP, enhanced green fluorescent protein.

To verify whether repeated cell sorting and EGFP expression could alter the characteristics of the four cell types, frozen EGFP-expressing cells were thawed for observation of morphology and determination of selected protein expression, including three constitutively expressed proteins (FAK, Bcl-xL, PTEN) and four cell type–specific proteins (CK13, Thy-1, ZO-1, α-SMA). The results revealed that repeated cell sorting and transduction with EGFP-encoding pseudovirions did not alter cell morphology (Fig 4). However, FAK was suppressed and Thy-1 was enhanced in EGFP-positive HCFs, but not in the other cell types (Fig 5).

### Ocular cells exhibit differential sensitivity to puromycin

**Fig 6:**
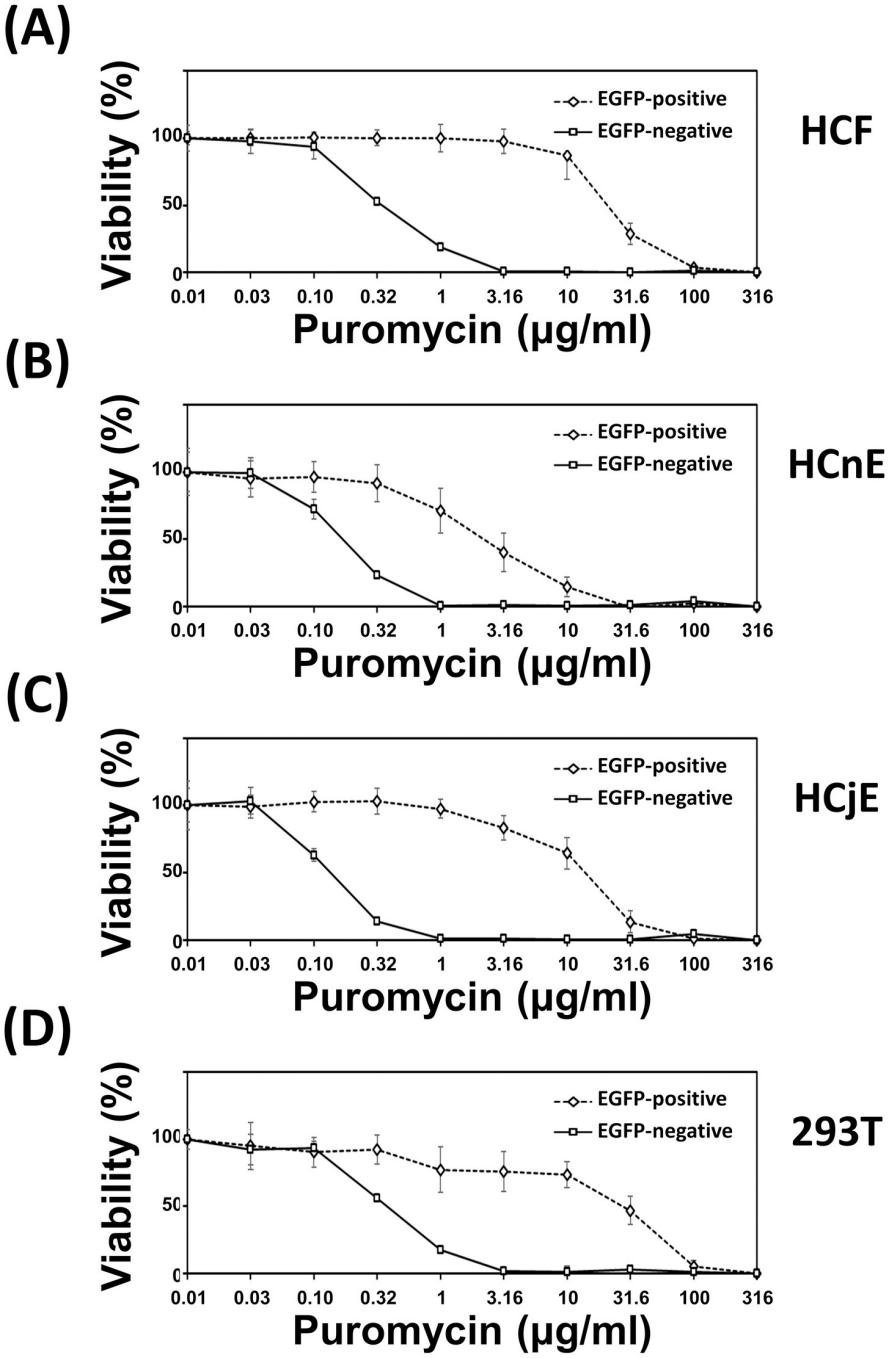
Tolerance to puromycin of the sorted EGFP-expressing cells. All EGFP-positive and EGFP-negative cells were subjected to repeated cell sorting to achieve >95% purity. Each cell type was seeded into individual wells of a 96-well plate: (A) 2.0 × 10^3^ HCFs, (B) 2.0 × 10^4^ HCnEs, (C) 2.0 × 10^4^ HCjEs, and (D) 2.0 × 10^4^ HEK-293T cells. At 24 h post-seeding, the medium was refreshed, and both EGFP-positive and EGFP-negative cells were incubated with a series of 3.16-fold dilutions of puromycin ranging from 0.01 to 316 μg/ml. After incubation for 48 h, cell viability was analyzed with the CCK-8 reagent. Data are expressed as the mean ± S.D. from three independent experiments. The bars correspond to the S.D. of the mean.

The selection of cells with an antibiotic is an alternative to cell sorting for isolating EGFP-positive cells. Therefore, to assess any differences in antibiotic sensitivity among the ocular cell types with or without EGFP expression, the cells were incubated with puromycin (Fig 6 and S4 Fig). Cell viability curves were used to estimate the puromycin IC_50_ values for each cell type (Table 1 and S1 Table). EGFP-positive HCFs were most resistant to puromycin, whereas EGFP-positive HCnEs were least resistant. However, all the EGFP-negative cells were much more sensitive to puromycin, with all IC_50_ values being ≤1.00 μg/ml].

**Table 1:** IC_50_ values for puromycin among the sorted cells.

Cell	Population	IC_50_ (μg/ml)	R^2^ Value
HCFs	EGFP-positive	21.8	0.9999
	EGFP-negative	0.35	0.9978
HCnEs	EGFP-positive	2.32	0.9981
	EGFP-negative	0.16	0.9983
HCjEs	EGFP-positive	13.1	0.9925
	EGFP-negative	0.12	0.9973
HEK-293T cells	EGFP-positive	39.3	0.9663
	EGFP-negative	0.38	0.997

## Discussion

Efficient expression of transgenes is critical for researchers not only to elucidate the potential function of a specific protein in mammalian cells but also to enhance the production of recombinant proteins [36]. In this regard, lentiviral transduction is an effective means for improving transgene expression in mammalian cells in terms of both stability and persistence [10, 37]. Using this lentiviral transduction system, we previously showed that several transgenes could be expressed in primary HCFs, *e*.*g*., EGFP alone and EGFP-fused paxillin [14], EGFP-fused ZO-1 [4], and matrix metalloproteinase-9 fused with EGFP driven by an internal ribosome entry site [3]. Our current results indicate that lentiviral EGFP expression in ocular cells was most difficult in HCFs, followed by HCnEs and HCjEs (Fig 1). Only 48.0% of HCFs were EGFP-positive, even though 5.0 × 10^6^ R.T.U. of pseudovirions was used to transduce 2.0 × 10^5^ primary HCFs, implying a 25-fold excess of pseudovirions over HCFs, which constitutes a relatively high titer (Fig 1A). These results indicate that it is quite difficult to express transgenic proteins in primary HCFs.

The high titer of EGFP pseudovirions would theoretically increase not only the number of the EGFP-positive cells but also the cellular abundance of EGFP. Indeed, the mean fluorescence intensity for each cell type that had been transduced with pseudovirions at 5.0 × 10^6^ R.T.U. was higher than that at 1.0 × 10^6^ R.T.U. pseudovirions (Fig 1E and S1 Fig). However, infection with 5.0 × 10^6^ R.T.U. pseudovirions did not result in a five-fold increase in the percentage of EGFP-positive cells compared with infection with 1.0 × 10^6^ R.T.U. pseudovirions. This may be a consequence of the fact that HCFs have the largest relative cell volume (Fig 4). A total of 2.0 × 10^5^ HCFs had to be seeded into the 10-cm culture dish to achieve confluency and to carry out the experiments for Fig 1. In contrast, 10 times more cells, *i*.*e*., 2.0 × 10^6^ cells, were required for HCnEs, HCjEs, and HEK-293T cells for the same experiments. The binding of pseudoviral particles to plasma-membrane receptors is required for infection of target cells. Therefore, a larger surface area, higher receptor density, and greater binding affinity could all contribute to a higher infection rate. The surface area-to-volume ratio decreases as cell volume increases. The relatively larger HCFs in our study were less easily infected by lentivirus because this ratio was smaller relative to the ratios of the other cell types. However, the density of lentiviral receptors may differ among ocular cell types, and this aspect must be addressed in the future.

Among current techniques, cell sorting is the most effective for facilitating the isolation of homogeneous cell populations by avoiding antibiotic selection [23]. Therefore, cell sorting has been commonly adopted to isolate target cells [24–26, 38]. Indeed, we previously used high-performance cell sorting to increase the homogeneity of transgenic HCFs [3]. However, the initial results were suboptimal with respect to the efficiency and persistence of transgene expression in primary ocular cells. Therefore, as demonstrated in our current study, we developed a more effective cell-sorting process with single-cell mode for efficient isolation of a homogeneous population of EGFP-positive HCFs (Figs 1A and 2A).

In mammalian cells, chromosomes are inherited in a more stable manner than are plasmids [39], so lentiviral transduction is an efficient method of expressing transgenic proteins in mammalian cells owing to the stable integration of the transgene to maintain the potency of its expression [9]. Also, cell sorting is expected to separate various EGFP-positive cell types to yield homogeneous populations [23]. However, regardless of the cell type we used in our present study, the cells were passaged twice before the initial sorting of EGFP-positive cells, and EGFP expression was reduced significantly after the second sorting (Fig 2 and S2 Fig). This revealed that the efficiency of pseudovirion-mediated transduction was reduced upon sorting of the EGFP-positive cells. However, cell sorting is still a more efficient approach than antibiotic selection for isolating homogeneous populations of EGFP-positive cells. Furthermore, the EGFP-positive cells remained >95% pure after sequential repeated culture under cell type–specific cultivation conditions even after storage in liquid nitrogen (data not shown).

During the cell sorting process, all the EGFP-transduced cells were gated in the same defined regions of the FSC-SSC plots to enable collection of cells that were homogeneous in size and morphology. During the repeated cell sorting and subsequent culture steps, the morphology and fluorescence intensity of the sorted cells were also assessed daily (Fig 4). Both HCFs and myofibroblasts expressed Thy-1, whereas only myofibroblasts expressed α-SMA [29]. Transduction with EGFP-encoding pseudovirions and subsequent cell sorting enhanced Thy-1 expression in isolated HCFs, whereas transduction did not alter α-SMA expression. These results suggested that transduction with pseudovirions promoted reversion to a more fibroblast-like state for the primary corneal stromal cells (Fig 5). However, sorting did not alter the expression of any other protein except Thy-1. In addition, EGFP expression and repeated sorting did not alter the morphology or protein expression pattern of HCnEs or HCjEs. These results further suggest that up to four rounds of cell sorting are sufficient to yield homogeneous populations of EGFP-positive ocular cells with >95% persistence without significant alterations in cell morphology or cell-specific characteristics.

Regarding antibiotic selection, our results reveal that no specific concentration of puromycin can completely differentiate between homogeneous populations of EGFP-positive and EGFP-negative cells. At the selection concentration of 1 μg/ml for puromycin, the respective viabilities of EGFP-negative and EGFP-positive were 18.7% and 99.9% for HCFs, 0.5% and 71.2% for HCnEs, 1.1% and 97.5% for HCjEs, and 17.6% and 77.4% in HEK-293T cells (Fig 6 and S4 Fig). The IC_50_ values indicated that ocular cells differ with respect to puromycin tolerance, *i*.*e*., primary HCFs > HCjEs > HCnEs (Table 1 and S1 Table). Also, recent studies have demonstrated that traditional selection using antibiotics to isolate gene-positive transformants might cause growth defects and thereby lead to retention of the unstable plasmid-carrying cells [22, 40]. Puromycin inhibits protein synthesis by ribosome-catalyzed incorporation into the C-terminus of elongating nascent chains, blocking further extension and resulting in premature termination of translation [41]. Cell types that have a distinct gene-expression profile tend to be more sensitive to puromycin [42]. Therefore, compared with fibroblasts, differentiated epithelial cells may be more sensitive to puromycin. Among the cells we tested, HCFs and HEK-293T cells share one similarity, *i*.*e*., mesenchymal characteristics. HCFs differentiate from keratocytes, which are derived from mesenchymal cells [43], whereas HEK-293T cells express both epithelial and mesenchymal cell-adhesion molecules [44]. **Still, these two cell types** exhibited similar puromycin resistance in both their EGFP-negative and EGFP-positive forms. Our results showed that the IC_50_ values for EGFP-negative HCFs and EGFP-negative HEK-293T cells were 0.35 μg/ml and 0.38 μg/ml, respectively (Table 1 and S1 Table). Both were higher than the 0.16 μg/ml calculated for EGFP-negative HCnEs and 0.12 μg/ml for EGFP-negative HCjEs. Similarly, the IC_50_ values for EGFP-positive HCFs and EGFP-positive HEK-293T cells were 21.8 μg/ml and 39.3 μg/ml, respectively. These were also higher than the 2.32 μg/ml for EGFP-positive HCnEs and 13.1 μg/ml for EGFP-positive HCjEs. These data suggest that repeated cell sorting is superior to antibiotic selection for purifying populations of cells expressing a specific transgene(s).

Our results also revealed that multi-sorted EGFP-negative cells grew faster than multi-sorted EGFP-positive cells during puromycin-free cultivation (Fig 3). This would lead to a progressively larger proportion of EGFP-negative cells and result in a net decrease in the percentage of EGFP-expressing cells during subsequent passages. Random integration of lentiviral EGFP-expressing sequences in the host genome might also result in a population of unstable EGFP-positive cells, which would gradually vanish during antibiotic-free cultivation. Therefore, our repeated cell-sorting process is conducive to the harvesting of a homogeneous population of stable EGFP-positive cells.

## Conclusions

A higher proportion of cells expressing target genes is desirable for experiments, but it is usually difficult to deliver a transgene to ocular cells. This study mainly demonstrates the efficiency of isolating a homogeneous population of transgene-expressing ocular cells with a fluorescent reporter using up to four rounds of cell sorting. We stored pure sorted EGFP-positive cells in liquid nitrogen, with subsequent thawing, cultivation, and analysis by flow cytometry. The high level of purity was maintained even after storage and repeated cell sorting to achieve >95% EGFP-positive cells in two consecutive passages. Therefore, we suggest that a combination of lentivirus-based delivery and repeated cell sorting is a useful approach for improving and maintaining a persistent and homogeneous population of cells expressing a fluorescent protein.

## Supporting information

S1 Fig Signal intensity of each EGFP-transduced cell type as assessed with a cell sorter using the FL1 channel. All the EGFP-transduced cells were analyzed by a cell sorter, and the signal intensity of the gated EGFP-positive cells was determined in the FL1 channel. Original numbers in mean, median, and mode were calculated and analyzed with the Student’s *t*-test. Shown is the proportion of EGFP-positive cells transduced with 5.0 × 10^6^ R.T.U. vs 1.0 × 10^6^ R.T.U. as assessed by the intensity in the FL1 channel.
(PDF)

S2 Fig The original proportion of the EGFP-positive cells during cell sorting at the indicated rounds. (PDF)

S3 Fig Growth of the sorted EGFP-negative and EGFP-positive cells. Cell viability of the sorted EGFP-negative cells and EGFP-positive was analyzed at the indicated time points with the CCK-8 reagent.
(PDF)

S4 Fig Viability of the sorted EGFP-negative and EGFP-positive cells cultured in the presence of puromycin. The viability (CCK-8 reagent) of the EGFP-positive and EGFP-negative cells was assessed after incubation with a series of 3.16-fold dilutions of puromycin ranging from 0.01 to 316 μg/ml.
(PDF)

S1 Table Calculation of IC_50_ values for the sorted cells that had been exposed to puromycin. (PDF)
