# Supplementary material for: Repeated cell sorting ensures the homogeneity of ocular cell populations expressing a transgenic protein
Source: PLoS One. 2022 Mar 25;17(3):e0265183. doi: 10.1371/journal.pone.0265183 (PMC8956163; doi:10.1371/journal.pone.0265183)
Supplement: S1 Fig — All the EGFP-transduced cells were analyzed by a cell sorter, and the signal intensity of the gated EGFP-positive cells was determined in the FL1 channel. Original numbers in mean, median, and mode were calculated and analyzed with the Student’s t-test. Shown is the proportion of EGFP-positive cells transduced with 5.0 × 10^6^ R.T.U. vs 1.0 × 10^6^ R.T.U. as assessed by the intensity in the FL1 channel. (PDF) [file pone.0265183.s001.pdf]

Intensity in FL1 channel						
**HCF**	1.0 x 10^6 R.T.U.			5.0 x 10^6 R.T.U.		
	Mean	Median	Mode	Mean	Median	Mode
1	1314.6	508.0	581.7	2972.7	1145.1	1435.0
2	1170.4	443.7	834.8	3177.1	1253.3	2358.0
3	1270.9	485.6	387.5	3112.8	1253.3	1311.1

➔

Calculation						
**HCF**	1.0 x 10^6 R.T.U.			5.0 x 10^6 R.T.U.		
	Mean	Median	Mode	Mean	Median	Mode
Average	1252.0	479.1	601.3	3087.5	1217.2	1701.4
SD	73.9	32.7	224.3	104.5	62.5	572.0
T-test				0.000	0.000	0.032
Fold				2.47	2.54	2.83

Intensity in FL1 channel						
**HCnE**	1.0 x 10^6 R.T.U.			5.0 x 10^6 R.T.U.		
	Mean	Median	Mode	Mean	Median	Mode
1	599.0	215.4	119.8	885.9	338.4	387.5
2	614.7	235.8	150.1	966.6	323.5	323.5
3	651.6	225.4	196.8	937.1	338.4	309.2

➔

Calculation						
**HCnE**	1.0 x 10^6 R.T.U.			5.0 x 10^6 R.T.U.		
	Mean	Median	Mode	Mean	Median	Mode
Average	621.8	225.5	155.6	929.8	333.4	340.0
SD	27.0	10.2	38.8	40.8	8.6	41.7
T-test				0.000	0.000	0.003
Fold				1.50	1.48	2.19

Intensity in FL1 channel						
**HCjE**	1.0 x 10^6 R.T.U.			5.0 x 10^6 R.T.U.		
	Mean	Median	Mode	Mean	Median	Mode
1	973.4	387.5	762.7	2382.4	1000.0	2059.3
2	969.3	405.4	531.5	2486.6	1095.0	1968.4
3	979.9	387.5	729.0	2402.5	1046.2	834.8

➔

Calculation						
**HCjE**	1.0 x 10^6 R.T.U.			5.0 x 10^6 R.T.U.		
	Mean	Median	Mode	Mean	Median	Mode
Average	974.2	393.4	674.4	2423.9	1046.9	1620.8
SD	5.3	10.3	124.9	55.3	47.3	682.3
T-test				0.000	0.001	0.067
Fold				2.66	2.40	2.49

Intensity in FL1 channel						
**293T**	1.0 x 10^6 R.T.U.			5.0 x 10^6 R.T.U.		
	Mean	Median	Mode	Mean	Median	Mode
1	5924.7	4052.6	4052.6	9474.2	6085.8	6968.5
2	6186.8	4240.8	3874.7	9710.9	6366.8	7979.2
3	6398.5	4436.7	4053.6	10076.6	6660.9	7979.2

➔

Calculation						
**293T**	1.0 x 10^6 R.T.U.			5.0 x 10^6 R.T.U.		
	Mean	Median	Mode	Mean	Median	Mode
Average	6170.0	4243.4	3993.6	9753.9	6371.1	7642.3
SD	237.4	192.0	103.0	303.5	287.6	583.6
T-test				0.000	0.000	0.004
Fold				1.58	1.50	1.91
