# Supplementary material for: Repeated cell sorting ensures the homogeneity of ocular cell populations expressing a transgenic protein
Source: PLoS One. 2022 Mar 25;17(3):e0265183. doi: 10.1371/journal.pone.0265183 (PMC8956163; doi:10.1371/journal.pone.0265183)
Supplement: S2 Fig — (PDF) [file pone.0265183.s002.pdf]

HCF

No.	1st Sorting	2nd Sorting	3rd Sorting	4th Sorting	Final Analysis
1	13.7%	77.1%	97.5%	98.4%	99.1%
2	14.0%	91.8%	99.1%		99.5%
3	40.1%	95.0%	97.5%		98.4%
4	37.9%	93.6%	97.5%		96.5%
5	23.4%	95.2%			96.8%
6	41.4%	88.6%	95.5%		97.4%
7	75.5%	93.7%	98.4%		99.2%
8	76.4%	91.1%	98.4%		98.3%
9	48.0%	77.8%	92.5%	97.8%	99.6%
Average	41.2%	89.3%	97.0%	98.1%	
SD	23.1%	7.0%	2.1%	0.4%	
Median	40.1%	91.8%	97.5%	98.1%	
Number (n)	9	9	8	2	

HCnE

No.	1st Sorting	2nd Sorting	3rd Sorting	4th Sorting	Final Analysis
1	45.8%	78.1%	97.6%	98.0%	98.3%
2	52.1%	82.1%	95.1%	97.5%	96.9%
3	59.4%	64.8%	88.3%	96.1%	95.9%
4	64.2%	87.0%	96.2%	98.8%	97.8%
5	43.2%	52.7%	83.6%	96.2%	97.9%
6	43.2%	52.7%	83.6%	95.9%	98.9%
Average	51.3%	69.6%	90.8%	97.1%	
SD	8.9%	15.0%	6.4%	1.2%	
Median	49.0%	71.4%	91.7%	96.8%	
Number (n)	6	6	6	6	

HCjE

No.	1st Sorting	2nd Sorting	3rd Sorting	4th Sorting	Final Analysis
1	87.6%	95.5%	98.1%		99.1%
2	52.9%	91.3%	97.3%		97.3%
3	57.8%	89.3%	96.2%	98.2%	98.7%
4	43.3%	81.2%	92.7%	95.2%	96.4%
5	32.6%	52.4%	87.8%	92.1%	95.9%
6	19.6%	38.1%	90.4%	96.2%	97.2%
Average	49.0%	74.6%	93.8%	95.4%	
SD	23.5%	23.7%	4.1%	2.5%	
Median	48.1%	85.2%	94.5%	95.7%	
Number (n)	6	6	6	4	

293T

No.	1st Sorting	2nd Sorting	3rd Sorting	4th Sorting	Final Analysis
1	69.9%	95.5%	95.3%		95.5%
2	79.2%	98.2%	99.1%		98.9%
3	54.4%	92.3%	97.3%		95.5%
4	49.2%	93.2%	97.9%		98.3%
5	72.9%	98.3%	98.6%		99.0%
Average	65.1%	95.5%	97.6%		
SD	12.8%	2.8%	1.5%		
Median	69.9%	95.5%	97.9%		
Number (n)	5	5	5	0	
