# Supplementary material for: Repeated cell sorting ensures the homogeneity of ocular cell populations expressing a transgenic protein
Source: PLoS One. 2022 Mar 25;17(3):e0265183. doi: 10.1371/journal.pone.0265183 (PMC8956163; doi:10.1371/journal.pone.0265183)
Supplement: S3 Fig — Cell viability of the sorted EGFP-negative cells and EGFP-positive was analyzed at the indicated time points with the CCK-8 reagent. (PDF) [file pone.0265183.s003.pdf]

Determination using CCK-8 reagent

HCF	No.	OD450-650		
		D0	D1	D2
EGFP-positive	1	0.471	0.996	3.310
	2	0.441	1.217	2.497
	3	0.435	1.239	2.927
EGFP-negative	1	0.178	0.494	1.229
	2	0.163	0.563	1.633
	3	0.172	0.432	1.483

Base	D0	D1	D2
0.449	1.049	2.218	7.372
0.449	0.982	2.710	5.561
0.449	0.969	2.759	6.519
0.171	1.041	2.889	7.187
0.171	0.953	3.292	9.550
0.171	1.006	2.526	8.673

HCF	Value	Folds		
		D0	D1	D2
EGFP-positive	Average	1.000	2.563	6.484
	SD	0.043	0.299	0.906
EGFP-negative	Average	1.000	2.903	8.470
	SD	0.044	0.383	1.194
	T-test		0.146	0.042

HCnE	No.	OD450-650		
		D0	D1	D2
EGFP-positive	1	0.242	0.438	1.893
	2	0.256	0.522	1.543
	3	0.245	0.567	1.888
EGFP-negative	1	0.119	0.267	1.247
	2	0.148	0.357	1.314
	3	0.125	0.428	1.013

Base	D0	D1	D2
0.248	0.977	1.769	7.643
0.248	1.034	2.108	6.230
0.248	0.989	2.289	7.623
0.131	0.911	2.043	9.543
0.131	1.133	2.732	10.056
0.131	0.957	3.276	7.753

HCnE	Value	Folds		
		D0	D1	D2
EGFP-positive	Average	1.000	2.055	7.166
	SD	0.030	0.264	0.810
EGFP-negative	Average	1.000	2.684	9.117
	SD	0.117	0.617	1.209
	T-test		0.090	0.040

HCjE	No.	OD450-650		
		D0	D1	D2
EGFP-positive	1	0.556	1.265	3.215
	2	0.660	1.562	3.607
	3	0.742	1.603	3.511
EGFP-negative	1	0.236	0.534	1.766
	2	0.244	0.588	1.609
	3	0.280	0.724	1.416

Base	D0	D1	D2
0.653	0.852	1.938	4.926
0.653	1.011	2.393	5.527
0.653	1.137	2.456	5.379
0.253	0.932	2.108	6.971
0.253	0.963	2.321	6.351
0.253	1.105	2.858	5.589

HCjE	Value	Folds		
		D0	D1	D2
EGFP-positive	Average	1.000	2.263	5.277
	SD	0.143	0.283	0.313
EGFP-negative	Average	1.000	2.429	6.304
	SD	0.093	0.386	0.692
	T-test		0.290	0.040

293T	No.	OD450-650		
		D0	D1	D2
EGFP-positive	1	0.493	1.113	2.372
	2	0.518	1.207	2.581
	3	0.512	1.428	2.876
EGFP-negative	1	0.213	0.676	1.885
	2	0.259	0.638	2.125
	3	0.264	0.578	1.658

Base	D0	D1	D2
0.508	0.971	2.192	4.672
0.508	1.020	2.378	5.084
0.508	1.009	2.813	5.665
0.245	0.868	2.755	7.683
0.245	1.056	2.601	8.662
0.245	1.076	2.356	6.758

293T	Value	Folds		
		D0	D1	D2
EGFP-positive	Average	1.000	2.461	5.141
	SD	0.026	0.319	0.499
EGFP-negative	Average	1.000	2.571	7.701
	SD	0.115	0.201	0.952
	T-test		0.320	0.007
