# Supplementary material for: Repeated cell sorting ensures the homogeneity of ocular cell populations expressing a transgenic protein
Source: PLoS One. 2022 Mar 25;17(3):e0265183. doi: 10.1371/journal.pone.0265183 (PMC8956163; doi:10.1371/journal.pone.0265183)
Supplement: S4 Fig — The viability (CCK-8 reagent) of the EGFP-positive and EGFP-negative cells was assessed after incubation with a series of 3.16-fold dilutions of puromycin ranging from 0.01 to 316 μg/ml. (PDF) [file pone.0265183.s004.pdf]

Determination using CCK-8 reagent

HCF	No.	µg/ml									
		0.01	0.0316	0.1	0.316	1	3.16	10	31.6	100	316
EGFP-positive	1	3.375	3.331	3.339	3.3	3.236	3.177	2.976	0.913	0.152	0.049
	2	3.438	3.433	3.418	3.415	3.413	3.319	2.726	1.032	0.173	0.039
	3	3.253	3.326	3.374	3.375	3.408	3.35	3.066	1.063	0.146	0.042
EGFP-negative	1	1.677	1.642	1.499	0.891	0.307	0.025	0.013	0.008	0.009	-0.002
	2	1.622	1.723	1.666	0.889	0.327	0.02	0.015	0.008	0.028	0.003
	3	1.721	1.542	1.536	0.874	0.317	0.021	0.021	0.016	0.043	0.014

Conc.	µg/ml									
	0.01	0.0316	0.1	0.316	1	3.16	10	31.6	100	316
Average	3.355	3.363	3.377	3.363	3.352	3.282	2.923	1.003	0.157	0.043
SD	0.094	0.060	0.040	0.058	0.101	0.092	0.176	0.079	0.014	0.005
Average	1.673	1.636	1.567	0.885	0.317	0.022	0.016	0.011	0.027	0.005
SD	0.050	0.091	0.088	0.009	0.010	0.003	0.004	0.005	0.017	0.008

HCnE	No.	µg/ml									
		0.01	0.0316	0.1	0.316	1	3.16	10	31.6	100	316
EGFP-positive	1	1.354	1.349	1.397	1.279	0.939	0.507	0.197	0.033	0.071	0.052
	2	1.661	1.478	1.487	1.475	1.24	0.705	0.329	0.071	0.122	0.075
	3	1.657	1.618	1.623	1.475	1.204	0.781	0.312	0.067	0.107	0.067
EGFP-negative	1	1.153	1.034	0.732	0.271	0.056	0.062	0.061	0.061	0.094	0.053
	2	1.094	1.145	0.851	0.294	0.066	0.071	0.056	0.075	0.103	0.054
	3	0.879	0.922	0.718	0.287	0.046	0.056	0.059	0.055	0.072	0.047

Conc.	µg/ml									
	0.01	0.0316	0.1	0.316	1	3.16	10	31.6	100	316
Average	1.557	1.482	1.502	1.410	1.128	0.664	0.279	0.057	0.100	0.065
SD	0.176	0.135	0.114	0.113	0.164	0.141	0.072	0.021	0.026	0.012
Average	1.042	1.034	0.767	0.284	0.056	0.063	0.059	0.064	0.090	0.051
SD	0.144	0.112	0.073	0.012	0.010	0.008	0.003	0.010	0.016	0.004

HCjE	No.	µg/ml									
		0.01	0.0316	0.1	0.316	1	3.16	10	31.6	100	316
EGFP-positive	1	3.056	2.799	2.891	2.913	2.741	2.325	1.772	0.346	0.07	0.061
	2	2.696	2.838	2.977	3.096	2.849	2.434	2.006	0.508	0.067	0.056
	3	2.926	2.961	3.042	2.946	2.875	2.502	1.885	0.452	0.093	0.058
EGFP-negative	1	1.619	1.587	1.027	0.265	0.063	0.059	0.057	0.054	0.104	0.04
	2	1.766	1.686	1.1	0.291	0.064	0.061	0.048	0.041	0.121	0.049
	3	1.516	1.788	1.011	0.237	0.059	0.051	0.044	0.046	0.116	0.046

Conc.	µg/ml									
	0.01	0.0316	0.1	0.316	1	3.16	10	31.6	100	316
Average	2.893	2.866	2.970	2.985	2.822	2.420	1.888	0.435	0.077	0.058
SD	0.182	0.085	0.076	0.098	0.071	0.089	0.117	0.082	0.014	0.003
Average	1.634	1.687	1.046	0.264	0.062	0.057	0.050	0.047	0.114	0.045
SD	0.126	0.101	0.047	0.027	0.003	0.005	0.007	0.007	0.009	0.005

293T	No.	µg/ml									
		0.01	0.0316	0.1	0.316	1	3.16	10	31.6	100	316
EGFP-positive	1	2.641	2.554	2.296	2.334	1.91	1.925	1.941	1.292	0.35	0.24
	2	2.653	2.682	2.518	2.492	2.214	2.062	1.97	1.356	0.4	0.275
	3	2.626	2.335	2.42	2.538	2.188	2.22	2.12	1.495	0.438	0.29
EGFP-negative	1	1.841	1.68	1.642	1.077	0.399	0.159	0.118	0.154	0.149	0.154
	2	1.724	1.564	1.701	1.051	0.433	0.152	0.112	0.186	0.169	0.114
	3	1.863	1.793	1.743	1.074	0.426	0.141	0.195	0.166	0.114	0.1

Conc.	µg/ml									
	0.01	0.0316	0.1	0.316	1	3.16	10	31.6	100	316
Average	2.640	2.524	2.411	2.455	2.104	2.069	2.010	1.381	0.396	0.268
SD	0.014	0.175	0.111	0.107	0.169	0.148	0.096	0.104	0.044	0.026
Average	1.809	1.679	1.695	1.067	0.419	0.151	0.142	0.169	0.144	0.123
SD	0.075	0.115	0.051	0.014	0.018	0.009	0.046	0.016	0.028	0.028

Calculation

HCF	EGFP-positive	Max-Min	3.312	3.312	3.312	3.312	3.312	3.312	3.312	3.312	3.312	3.312
		Min	0.043	0.043	0.043	0.043	0.043	0.043	0.043	0.043	0.043	0.043
		V-V0	3.312	3.320	3.334	3.320	3.309	3.239	2.879	0.959	0.114	0.000
		Viability	1.000	1.002	1.007	1.002	0.999	0.978	0.869	0.290	0.034	0.000
	EGFP-negative	Max-Min	1.668	1.668	1.668	1.668	1.668	1.668	1.668	1.668	1.668	1.668
		Min	0.005	0.005	0.005	0.005	0.005	0.005	0.005	0.005	0.005	0.005
		V-V0	1.668	1.631	1.562	0.880	0.312	0.017	0.011	0.006	0.022	0.000
		Viability	1.000	0.977	0.936	0.527	0.187	0.010	0.007	0.003	0.013	0.000

HCnE	EGFP-positive	Max-Min	1.493	1.493	1.493	1.493	1.493	1.493	1.493	1.493	1.493	1.493
		Min	0.065	0.065	0.065	0.065	0.065	0.065	0.065	0.065	0.065	0.065
		V-V0	1.493	1.417	1.438	1.345	1.063	0.600	0.215	-0.008	0.035	0.000
		Viability	1.000	0.949	0.963	0.901	0.712	0.402	0.144	-0.005	0.024	0.000
	EGFP-negative	Max-Min	0.991	0.991	0.991	0.991	0.991	0.991	0.991	0.991	0.991	0.991
		Min	0.051	0.051	0.051	0.051	0.051	0.051	0.051	0.051	0.051	0.051
		V-V0	0.991	0.982	0.716	0.233	0.005	0.012	0.007	0.012	0.038	0.000
		Viability	1.000	0.992	0.722	0.235	0.005	0.012	0.007	0.012	0.039	0.000

HCjE	EGFP-positive	Max-Min	2.834	2.834	2.834	2.834	2.834	2.834	2.834	2.834	2.834	2.834
		Min	0.058	0.058	0.058	0.058	0.058	0.058	0.058	0.058	0.058	0.058
		V-V0	2.834	2.808	2.912	2.927	2.763	2.362	1.829	0.377	0.018	0.000
		Viability	1.000	0.991	1.027	1.033	0.975	0.833	0.645	0.133	0.006	0.000
	EGFP-negative	Max-Min	1.589	1.589	1.589	1.589	1.589	1.589	1.589	1.589	1.589	1.589
		Min	0.045	0.045	0.045	0.045	0.045	0.045	0.045	0.045	0.045	0.045
		V-V0	1.589	1.642	1.001	0.219	0.017	0.012	0.005	0.002	0.069	0.000
		Viability	1.000	1.034	0.630	0.138	0.011	0.008	0.003	0.001	0.043	0.000

293T	EGFP-positive	Max-Min	2.372	2.372	2.372	2.372	2.372	2.372	2.372	2.372	2.372	2.372
		Min	0.268	0.268	0.268	0.268	0.268	0.268	0.268	0.268	0.268	0.268
		V-V0	2.372	2.255	2.143	2.186	1.836	1.801	1.742	1.113	0.128	0.000
		Viability	1.000	0.951	0.904	0.922	0.774	0.759	0.735	0.469	0.054	0.000
	EGFP-negative	Max-Min	1.687	1.687	1.687	1.687	1.687	1.687	1.687	1.687	1.687	1.687
		Min	0.123	0.123	0.123	0.123	0.123	0.123	0.123	0.123	0.123	0.123
		V-V0	1.687	1.556	1.573	0.945	0.297	0.028	0.019	0.046	0.021	0.000
		Viability	1.000	0.923	0.932	0.560	0.176	0.017	0.011	0.027	0.013	0.000
