# Supplementary material for: Repeated cell sorting ensures the homogeneity of ocular cell populations expressing a transgenic protein
Source: PLoS One. 2022 Mar 25;17(3):e0265183. doi: 10.1371/journal.pone.0265183 (PMC8956163; doi:10.1371/journal.pone.0265183)
Supplement: S1 Table — (PDF) [file pone.0265183.s005.pdf]

Sigmoidal, 4PL, X is concentration	HCF_P	HCF_N	HCnE_P	HCnE_N	HCjE_P	HCjE_N	293T_P	293T_N
**Best-fit values**								
Bottom	0.1358	0.3391	-1.088	0.8404	-2.672	1.028	-19.17	1.072
Top	100	101.2	97.89	101.4	99.92	103.6	92.12	97.88
IC50	21.79	0.357	2.324	0.16	13.09	0.1239	39.29	0.3841
HillSlope	-2.407	-1.655	-1.215	-1.976	-1.612	-2.237	-0.8998	-1.758
logIC50	1.338	-0.4474	0.3663	-0.7958	1.117	-0.9069	1.594	-0.4155
Span	99.9	100.8	98.97	100.6	102.6	102.5	111.3	96.81
**Goodness of Fit**								
Degrees of Freedom	6	6	6	6	6	6	6	6
R squared	0.9999	0.9978	0.9981	0.9983	0.9925	0.9973	0.9663	0.997
Sum of Squares	2.423	39.58	32.47	28.72	131.6	44.39	403.2	51.74
Sy.x	0.6355	2.569	2.326	2.188	4.683	2.72	8.198	2.937
**Number of points**								
# of X values	10	10	10	10	10	10	10	10
# Y values analyzed	10	10	10	10	10	10	10	10

**Formula**	Y=Bottom + (Top-Bottom)/(1+(IC50/X)^HillSlope)

Verificaation								
**Conc. (µg/ml)**								
0.01	100.0	100.9	97.8	101.0	99.9	103.2	92.1	97.7
0.03	100.0	99.4	97.4	97.5	99.9	99.0	91.9	96.7
0.10	100.0	90.3	95.8	72.9	99.9	64.4	91.6	89.6
0.32	100.0	55.8	89.8	21.6	99.7	12.3	90.7	57.7
1.00	99.9	15.9	71.7	3.5	98.3	2.0	88.2	16.3
3.16	99.1	3.0	39.3	1.1	90.5	1.1	81.7	3.4
10.00	86.7	0.7	13.3	0.9	59.6	1.0	67.0	1.4
31.60	29.1	0.4	2.9	0.8	17.3	1.0	41.9	1.1
100.00	2.6	0.3	-0.1	0.8	1.1	1.0	14.4	1.1
316.00	0.3	0.3	-0.8	0.8	-2.1	1.0	-4.4	1.1
